# Mixed reality-supported near-infrared photoimmunotherapy for oropharyngeal cancer: a case report

**DOI:** 10.1097/MS9.0000000000002366

**Published:** 2024-08-07

**Authors:** Ryuhei Okada, Taku Ito, Hiroaki Kawabe, Takeshi Tsutsumi, Takahiro Asakage

**Affiliations:** a Department of Head and Neck Surgery, Tokyo Medical and Dental University; b Otorhinolaryngology, Tokyo Medical and Dental University, Tokyo, Japan

**Keywords:** Alluminox, case report, head and neck cancer, head-mounted display (HMD), IR700, mixed reality, photodynamic therapy

## Abstract

**Introduction and importance:**

Near-infrared photoimmunotherapy (NIR-PIT, Alluminox) uses an antibody-photoabsorber conjugate and light excitation, requiring precise illumination. Mixed reality (MR) technology can enhance medical procedures through advanced visualization and planning.

**Case presentation:**

An 86-year-old man with recurrent oropharyngeal cancer and right cervical metastasis received NIR-PIT. Three-dimensional models from computed tomography (CT) and FDG-PET/CT images were used as holograms on a head-mounted display (HMD) for precise light targeting.

**Clinical discussion:**

HMD-MR technology was utilized for preoperative simulation and guided ideal light direction during surgery. This improved the effectiveness of NIR-PIT.

**Conclusion:**

Three months post-treatment, no residual lesion was observed, demonstrating the utility of HMD-MR technology in optimizing NIR-PIT outcomes.

## Introduction

Highlights

- Case report demonstrating the efficacy of near-infrared photoimmunotherapy (NIR-PIT) for oropharyngeal cancer.
- Novel approach using mixed reality (MR) head-mounted display for ideal light illumination during NIR-PIT.
- Preoperative simulation and intraoperative MR guidance enabled ideal light delivery to the tumor.
- MR technology is useful for three-dimensional (3D) visualization, surgical planning, and assisting complex procedures.

Near-infrared photoimmunotherapy (NIR-PIT, Alluminox) is a molecular-targeted therapy that involves the use of an antibody-photoabsorber conjugate (APC) and an excitation light^1^. The photoabsorber used in NIR-PIT is IRDye700DX (IR700), which is activated with a light of 690 nm wavelength. NIR-PIT targeting epidermal growth factor receptor (EGFR) with cetuximab sarotalocan (cetuximab-IR700 conjugate, Akalux, Rakuten Medical Inc.) and a laser system (BioBlade, Rakuten Medical Inc.) has been used for treating patients with inoperable head and neck cancer in Japan since 2021, based on the results of clinical trials^2,3^. APC is administered intravenously to the patient, and the tumor is illuminated on the following day. Light is delivered with a frontal diffuser and/or cylindrical diffuser through a catheter; a frontal diffuser emits light frontally from the tip and is usually used for superficial tumors, while a cylindrical diffuser emits light cylindrically from several centimeters behind the tip (2, 3, or 4 cm), and is usually used for tumors located deeper from the surface. Thorough simulation tailored to the three-dimensional (3D) morphology of the tumor is crucial to ensure the delivery of light of sufficient intensity for effective treatment, as APC exerts no cytotoxicity in the absence of light exposure.

Recently, the medical field has witnessed the application of mixed reality (MR) technology, particularly in the fields of surgical education, simulation, and navigation^4,5^. Augmented reality (AR), a concept involving the integration of digital information with the user’s real-time environment, serves as its foundation. An extension of AR, known as MR, enables the interaction of real and virtual elements within an environment. MR allows for simultaneous experiencing of the physical world and virtual reality by overlaying images within the coordinate spaces. Through the use of a head-mounted display (HMD), surgeons can perform hands-free procedures while visualizing 3D computer graphic models (holograms) of each patient using intraoperative MR techniques. In this study, we report the case of a patient with oropharyngeal cancer who was successfully treated by cetuximab NIR-PIT with the use of a MR head-mounted display (HMD-MR). This study was conducted with the approval of the research ethics committee of Tokyo Medical and Dental University (M2018-086).

## Case presentation

This case report is reported in line with the SCARE 2023 criteria, Supplemental Digital Content 1, http://links.lww.com/MS9/A571 ^6^.

### Patient profile

**Figure 1:**
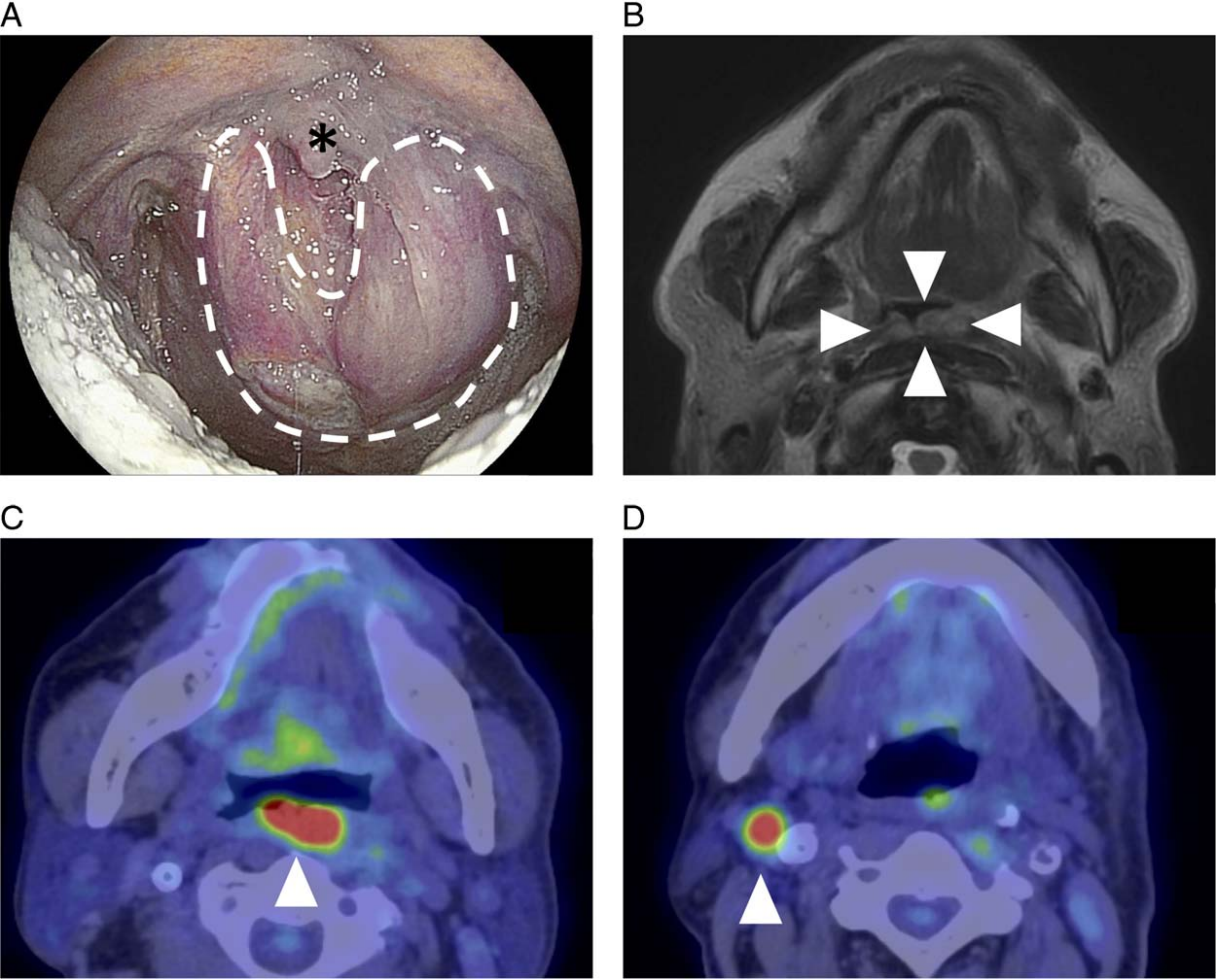
Preoperative images. (A) Endoscopic examination. The precise extent and outline of the tumor are obscure. A heart-shaped tumor (dotted line) is seen. *, uvula. (B) T2-weighted MRI imaging, a recurrent tumor (arrowheads) is seen in the oropharynx. (C, D) FDG-PET/computed tomography imaging. Recurrent lesions (arrowheads) are seen in the oropharynx (C) and right cervical lymph node (D).

An 86-year-old male was diagnosed as having recurrent oropharyngeal cancer. He had previously undergone endoscopic laryngo-pharyngeal surgery (ELPS) and boron neutron capture therapy (BNCT). He also had a history of laryngeal cancer that had been treated by total laryngectomy and radiation therapy. The patient presented with a tumor located in the posterior wall of the oropharynx and an enlarged right cervical lymph node (Fig. 1). Due to the overall compromised health of the patient, it was deemed that surgical resection with free flap reconstruction for the recurrent oropharyngeal cancer would be challenging. Consequently, NIR-PIT was selected as the preferred treatment approach. Prior to the NIR-PIT, a right neck dissection was performed.

### Creation of a 3D model and installation of the hologram on the HMD

**Figure 2:**
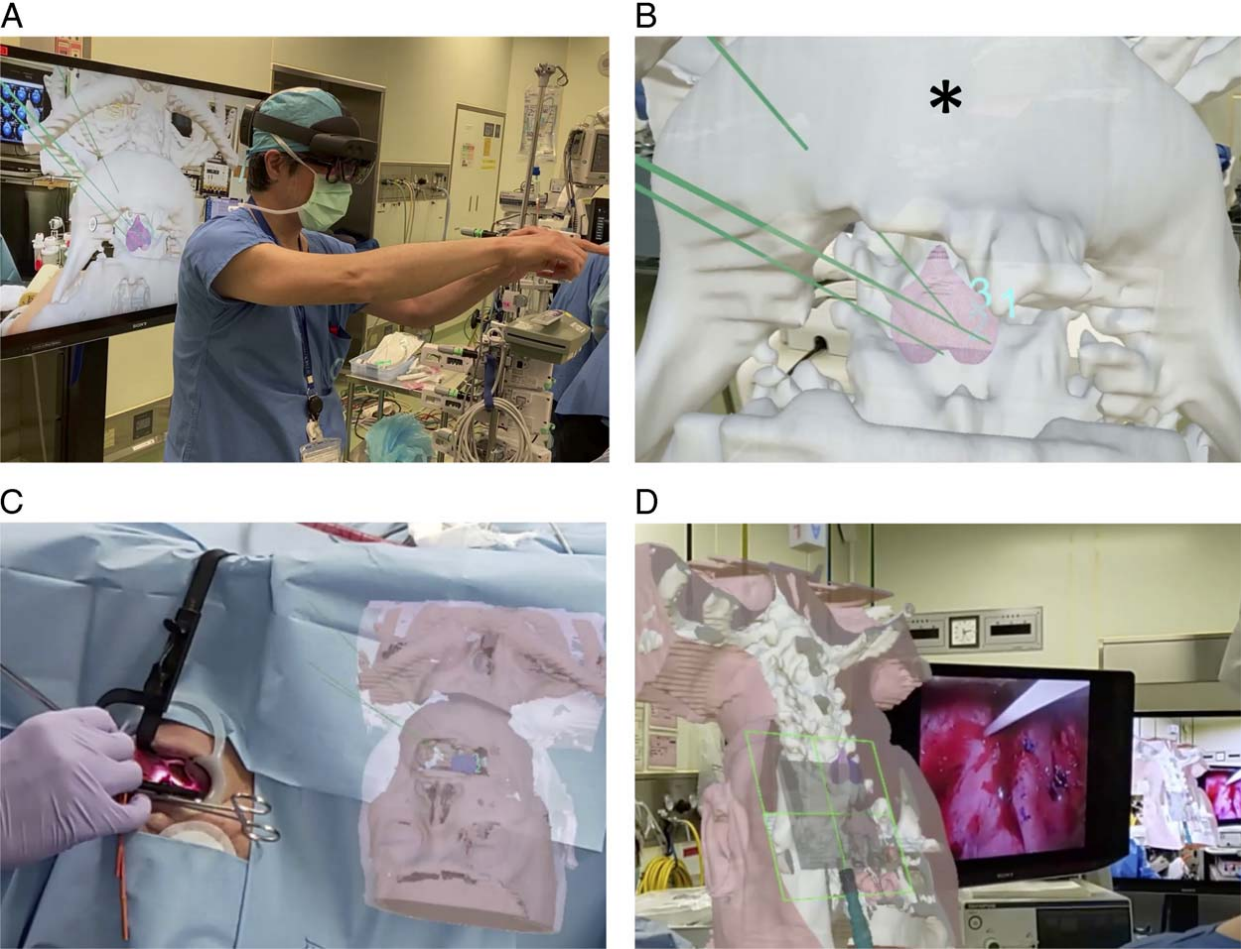
Holographic surgical guide through head-mounted display-mixed reality. (A, B) Preoperative simulation. The hologram was visualized with the head-mounted display. The hologram can be shared with other persons either through another head-mounted display or a two-dimensional monitor (A). The heart-shaped tumor was viewed as a three-dimensional hologram on the head-mounted display. *, mandible (B). (B) Illustrates the projected simulation of needle puncture paths within the context of the surrounding anatomical structures. It is important to note that these lines are conceptual in nature, designed to approximate the tumor’s spread and its relation to nearby landmarks such as the mandible. They do not depict the exact needle insertion paths utilized in the surgical procedure but serve as a guide for understanding potential surgical approaches and tumor extent. The actual needle insertion during surgery was performed with a high degree of precision and consideration to avoid vital structures, specifically tailored to the patient’s real-time anatomical configuration. (C, D) Intraoperative hologram. The surgeon wore HoloLens2 and displayed the virtual image adjacent to the real surgical field (C) or endoscopic monitor (D), allowing it to be visible simultaneously without the need to adjust the line of sight.

To effectively simulate the illumination procedure, we performed computed tomography (CT) imaging with the patient 3 days before the NIR-PIT in a mouth-open position, achieved by placing gauze in the mouth. From CT and FDG-PET/CT images, we then generated a customized 3D head and neck model specific to the patient. We processed the DICOM images in a 3D Slicer, using threshold segmentation to delineate the oropharyngeal lesion, bone, and a combined model of skin and mucosal boundaries, setting the thresholds to levels that encompass both skin and mucosal layers. We then converted the individual anatomical components into STL files, which were subsequently uploaded to Holoeyes XR (Holoeyes Inc.), an online service designed to facilitate the visualization of STL files on various wearable devices. After uploading, distinct anatomical parts were assigned specific colors or rendered translucent. The 3D virtual images were visualized as holograms on Microsoft HoloLens2 (Microsoft Corporation), and could be moved, rotated, and zoomed in through hand gestures (Fig. 2A). Furthermore, each hologram of the 3D virtual image could be adjusted to varying levels of translucency, allowing for a detailed exploration of the internal anatomical structures (Fig. 2B).

### Surgical procedure with holographic support

**Figure 3:**
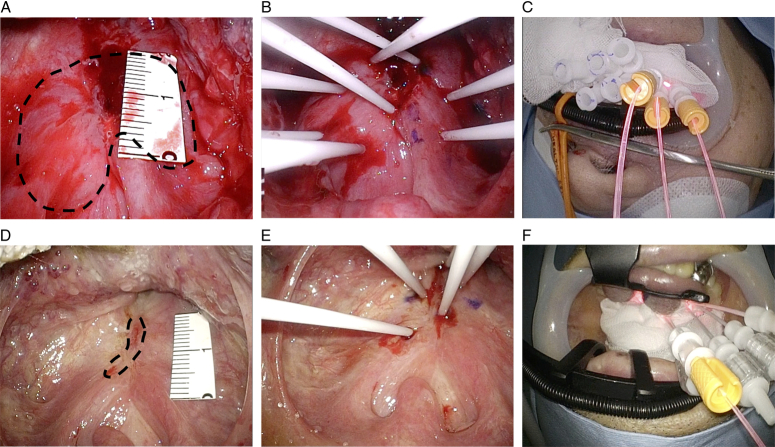
Endoscopic view during near-infrared photoimmunotherapy (NIR-PIT). (A–C) First NIR-PIT session. A heart-shaped tumor (dotted line) is observed in the oropharynx (A). Eight needle catheters were inserted into the tumor (B) and the tumor was illuminated through cylindrical diffusers (C). (D–F) Second NIR-PIT session. Only a small residual tumor (dotted line) is observed (D). Four needle catheters were inserted into the tumor (E) and the tumor was illuminated through cylindrical diffusers (F).

**Figure 4:**
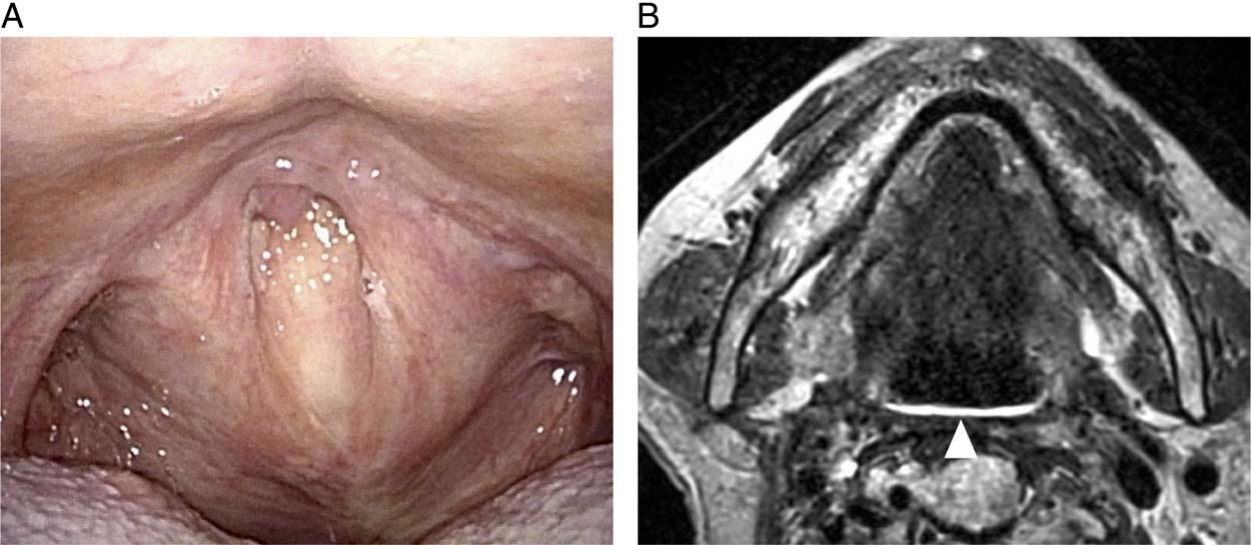
Postoperative images. (A) Endoscopic view. (B) T2-weighted MRI image. Arrowhead represents the place at which the tumor was previously located.

Cetuximab sarotalocan was intravenously administered, and the tumor was illuminated with the excitation light under general anesthesia on the following day. A mouth gag was placed and the tumor was observed by endoscopy and ultrasonography. The operators wore HoloLens2^TM^ in order to see both the real view and virtual images. We simulated ideal puncture positions and orientations of the needle catheters in the tumor and saved them as a holographic support (Fig. 2B, Supplementary Mov. 1, Supplemental Digital Content 2, http://links.lww.com/MS9/A572). Eight needle catheters were inserted into and around the tumor by referencing the holograms of the virtual images visualized through the HMD-MR (Figs. 2C,D, 3A,B, Supplementary Mov. 2, Supplemental Digital Content 3, http://links.lww.com/MS9/A573). Then, the tumor was illuminated with light through cylindrical diffusers inserted into the needle catheters. The tumor turned white in color immediately after the therapy (Fig. 3C). The patient had grade 1 local pain, but could resume oral intake from day one after the treatment. Mild pharyngeal edema was observed, which, however, gradually disappeared within a week. A residual tumor was suspected after the first NIR-PIT session, so that we planned a second treatment session. The second treatment was undertaken two months after the initial NIR-PIT session when only a small tumor remained. Four needle catheters were inserted into the tumor and the tumor was illuminated with light through cylindrical diffusers (Fig. 3D-F). The clinical course after the second treatment was similar to that after the first treatment. No recurrence was evident on endoscopy and MRI performed 3 months after the second session of NIR-PIT (Fig. 4).

## Discussion

We successfully performed NIR-PIT using the HMD-MR technology, which supports the integration of 3D holograms into the actual surgical environment. The HMD-MR technology was extremely beneficial for the planning and positioning during the catheter insertions, allowing for an ideal arrangement of the catheters and providing visual support with holograms close to the patient’s actual condition, alongside endoscopic imagery during the procedure.

To ensure delivery of a sufficient light intensity during NIR-PIT, it is essential to have a well-supported determination of the tumor’s extent and outline. Various devices have been explored to aid in precise light illumination through accurate catheter insertion. Ultrasonography, which was also used in our case, stands out as a valuable and easily accessible diagnostic tool for superficial tumors^7^. While ultrasonography provides real-time tumor images, it does not offer a clear view of the tumor’s 3D structure. Navigation systems offer advantages in locating and delineating various tumor types and are widely used in head and neck surgery^8^. However, conventional navigation systems have limitations, such as portability, high initial costs, and the need for separate 2D screens to track the current location. Intraoperative CT imaging can support the confirmation of catheter insertion, particularly for tumors located deep within tissues during NIR-PIT^9^, although limited availability and radiation exposure are its disadvantages. HMD-MR-assisted surgery is portable, cost-effective, and offers direct projection onto the actual surgical environment. This technology allows surgeons to perform surgery while continuously observing the operative field. In our case, the 3D model allowed us to visualize and simulate the insertion points, angles, and depths for the puncture lines more accurately. Another promising supporting tool for precise illumination during NIR-PIT is fluorescent imaging. The photoabsorber used in NIR-PIT (IR700) is a fluorescent dye that absorbs at 690 nm and emits 702 nm at the maximum. Its emission wavelength extends beyond 800 nm, making it detectable by commercially available cameras designed for indocyanine green (ICG), a widely used intraoperative imaging agent^10,11^.

We did not use the tumor hologram for navigation by overlaying it on the actual tumor, because the current accuracy of positioning using HMDs is not exceptionally high. Nguyen and colleagues reported a margin of error of ~10% in the horizontal dimension and up to 17% in the z dimension when measuring virtual objects indirectly through an HMD. In pursuit of greater accuracy, several studies have explored tool refinements^12^. Wu *et al.* ^13^ implemented an AR system that overlays 3D anatomical models onto patients through localization using superficial skin markers. Koyachi *et al.* ^14^ used registration markers affixed to an oral splint, allowing automatic alignment of the operative field and the models when recognized by the HoloLens device. Further research is warranted to explore the safety and efficacy of AR and MR for navigation, with a focus on their clinical applications: we consider these technologies as holding great promise for the future.

The patient initially presented with two recurrent tumors, one in the oropharynx and the other in the right cervical lymph node. We conducted a right neck dissection before initiating the NIR-PIT for the oropharyngeal recurrence. This strategy often proves beneficial for patients with multiple lesions, as it prioritizes resectable tumors before targeting unresectable ones, thus optimizing cancer control.

In conclusion, this report marks the first utilization of HMD-MR for NIR-PIT. Ideal light illumination is crucial for the effectiveness of NIR-PIT, and HMD-MR serves as a supportive tool during NIR-PIT.

## Ethical approval

Ethical approval for this study (Ethical Committee Number M2018-086) was provided by the Ethical Standards Committee of Tokyo Medical and Dental University, Tokyo, Japan on 28 August 2018.

## Consent

All procedures involving the patient described in the manuscript were conducted with informed consent, and the patient was made aware of the experimental nature of the treatment, the potential risks involved, and the use of mixed reality technology. Written informed consent was obtained from the patient for publication and any accompanying images. A copy of the written consent is available for review by the Editor-in-Chief of this journal on request.

## Source of funding

This manuscript was supported by JSPS KAKENHI Grant Number 23K14642.

## Author contribution

R.O. was responsible for the organization and coordination of the trial. T.I. was the chief investigator and responsible for the data analysis. R.O., T.I., H.K., T.T., and T.A. developed the trial design. All authors contributed to the writing of the final manuscript.

## Conflicts of interest disclosure

The authors declare that the research was conducted in the absence of any commercial or financial relationships that could be construed as a potential conflict of interest.

## Research registration unique identifying number (UIN)

1. Name of the registry: Monitorless exoscopic surgery using HMD and 3D holograms.
2. Unique Identifying number or registration ID: UMIN000047471.
3. Hyperlink to your specific registration (must be publicly accessible and will be checked):https://center6.umin.ac.jp/cgi-openbin/ ctr/ctr.cgi?function=brows&action=brows&recptno=R000054133&type=summary&language=J

## Guarantor

Taku Ito.

## Data availability statement

Not applicable.

## Provenance and peer review

Not applicable.

## Supplementary Material
